# Aurora B and Aurora C pools at two chromosomal regions collaboratively maintain chromosome alignment and prevent aneuploidy at the second meiotic division in mammalian oocytes

**DOI:** 10.3389/fcell.2024.1470981

**Published:** 2024-09-17

**Authors:** Anna Kouznetsova, Sonata Valentiniene, Jian-Guo Liu, Tomoya S. Kitajima, Hjalmar Brismar, Christer Höög

**Affiliations:** ^1^ Department of Cell and Molecular Biology, Karolinska Institutet, Stockholm, Sweden; ^2^ Laboratory for Chromosome Segregation, RIKEN Center for Biosystems Dynamics Research, Kobe, Japan; ^3^ Science for Life Laboratory, Department of Applied Physics, Royal Institute of Technology, Stockholm, Sweden

**Keywords:** Aurora B, Aurora C, meiosis, oocyte, second meiotic division, aneuploidy

## Abstract

Correct chromosome segregation is essential to preserve genetic integrity. The two protein kinases, Aurora B and its meiotic homolog Aurora C, regulate attachments between chromosomal kinetochores and microtubules, thereby contributing to the accuracy of the chromosome segregation process. Here we performed a detailed examination of the localization and activity of Aurora B/C kinases, their partner Incenp and the kinetochore target Hec1, during the second meiotic division in mouse oocytes. We found that a majority of Aurora B and C changed their localization from the outer kinetochore region of chromosomes at prometaphase II to an inner central region localized between sister centromeres at metaphase II. Depletion of the Aurora B/C pool at the inner central region using the haspin kinase inhibitor 5-iodotubercidin resulted in chromosome misalignments at the metaphase II stage. To further understand the role of the Aurora B/C pool at the central region, we examined the behaviour of single chromatids, that lack a central Aurora B/C pool but retain Aurora B/C at the outer kinetochores. We found that kinetochore-microtubule attachments at single chromatids were corrected at both prometaphase II and metaphase II stages, but that single chromatids compared to paired chromatids were more prone to misalignments following treatment of oocytes with the Aurora B/C inhibitory drugs AZD1152 and GSK1070916. We conclude that the Aurora B/C pool at the inner central region stabilizes chromosome alignment during metaphase II arrest, while Aurora B/C localized at the kinetochore assist in re-establishing chromosome positioning at the metaphase plate if alignment is lost. Collaboratively these two pools prevent missegregation and aneuploidy at the second meiotic division in mammalian oocytes.

## Introduction

The process of cell division, whether it be mitosis or meiosis, is a fundamental biological event that ensures the correct propagation of genetic material from one generation to the next. Serine-threonine kinase Aurora B and its meiosis-specific homolog Aurora C are key players in correction of kinetochore-microtubule attachments between chromosomes and spindle microtubules, thereby ensuring correct chromosome segregation between daughter cells (Nguyen and Schindler, 2017; Cairo and Lacefield, 2020).

Aurora B and Aurora C are key components of the Chromosomal Passenger Complex (CPC), along with Incenp, Borealin, and Survivin (Carmena et al., 2012). At late mitosis**,** Aurora B is predominantly localized at the inner centromere region located between sister chromatids. Loading of Aurora B at the inner centromere region is facilitated by haspin, a kinase that phosphorylates histone H3 on T3 (Wang et al., 2012). A second pool of Aurora B in mitotic cells has been identified on the outer centromere proximal to the kinetochores region, and a third pool at outer kinetochores about 20 nm outside of the localization of the CenpC protein (Broad et al., 2020; Liang et al., 2020).

Recruitment of Aurora B to the outer kinetochores and kinetochore-proximal region in mitotic cells takes place at the prometaphase stage, a majority Aurora B then become localized at the inner centromere region at the metaphase stage. Depletion of Aurora B from the centromere region or the kinetochore-proximal region did not result in chromosome segregation errors or reduced phosphorylation of Aurora B kinase kinetochore substrates, but when Aurora B was depleted from both regions simultaneously, chromosome segregation was compromised (Broad et al., 2020; Hadders et al., 2020; Liang et al., 2020). Aurora B localized at the outer kinetochore region has been shown to have a crucial role in preventing chromosome segregation errors when the central pools of Aurora B were depleted (Broad et al., 2020; Liang et al., 2020).

Meiosis consists of two cycles of chromosome segregation events. While there are similarities between mitosis and the second meiotic division (meiosis II), there are also many differences. The inter-kinetochore distance for sister chromatids is found to be roughly twice as large in meiosis II cells compared to mitotic cells (Magidson et al., 2011; Kouznetsova et al., 2019). Furthermore, while mitosis is completed in about 30 min, mouse and human oocytes can remain arrested at metaphase II for hours awaiting fertilization and formation of haploid cells (Schmidt et al., 2006). During the second meiotic division, Aurora B and Aurora C kinases are found on the kinetochores and at the inner region between sister chromatids (Kouznetsova et al., 2019)**.** Aurora B and C have been found to functionally regulate each other’s localization and activity in mouse oocytes (Nguyen et al., 2018)**.** It was shown that haspin kinase controls the inner centromeric localization of the Aurora B kinase through phophorylation of histone H3 on T3 in both MI and MII spermatocytes (Inés Berenguer et al., 2022) and that depletion of haspin kinase in mouse oocytes resulted in chromosome misalignments during the second meiotic division (Cairo et al., 2023). The exact roles of Aurora B/C during meiosis II in mammals, however, remain unclear.

In this study we set out to characterize the localization and activity of Aurora B/C kinases during the second meiotic division of mouse oocytes. Using super-resolution microscopy, we describe a change in localization of Aurora B and C from the kinetochore-proximal region at prometaphase II to the inner central region of sister chromosomes at metaphase II. To investigate the role of Aurora B/C kinases at the inner central region, we employed a chemical approach and inhibited haspin kinase in mouse oocytes using the small molecule inhibitor 5-iodotubercidin (5-Itu). We also examined the dynamics of single chromatids, which are distinct from normal chromosomes at the MII stage due to their lack of a paired sister chromatid. The single chromatids lack a central pool of Aurora B/C, making the single chromatids a valuable model in understanding the relative contribution of the different pools of Aurora B/C to chromosome alignment.

We find that Aurora B/C at the inner central region of chromosomes plays a critical role in maintaining chromosome alignment during the prolonged metaphase II stage, while Aurora B/C at the outer kinetochore contributes to re-establishing chromosomes alignment when lost at the prometaphase II and metaphase II stages of the second meiotic division.

## Materials and methods

### Mouse oocyte culture and microinjection

The animal experiments were approved by the Stockholm-North Animal Ethical Committee. Oocytes were taken from female mice carrying a null mutation for the *Sycp3* gene and their wild type siblings, produced on the mixed C57BL/6NCrl x 129/OlaIHsd background (Yuan et al., 2002). For immunolocalization experiments at the prometaphase II stage, oocytes were isolated from the ovaries at the germinal vesicle stage and cultured in M2 medium (Sigma) at 37°C; for immunolocalization experiments at the metaphase II stage oocytes were isolated from oviducts after PMS and hCG injections and transferred to KSOM media (Merck Millipore). To study the transition period between anaphase I onset and metaphase II arrest by time-lapse imaging in *Sycp3* ^*−/−*^oocytes, *in vitro* transcribed 2mEGFP-CENP-C or EGFP-CENP-C together with H2B-mCherry mRNA were microinjected at the germinal vesicle stage, oocytes were matured and imaged in M2 medium at 37°C as described in Kitajima et al. (2011). To study the chromosome behaviour at the metaphase II stage by time-lapse imaging, a reporter gene coding for histone H2B fused to mCherry was introduced into the experimental mouse strain by backcrossing with reporter mice carrying H2B-mCherry fusion gene (Abe et al., 2011). *In vitro* transcribed 2mEGFP-CENP-C mRNA or EGFP-CENP-C was microinjected into CSF-arrested oocytes expressing H2B-mCherry fusion protein. In inhibition assays, oocytes at the MII stage were cultured in M2 medium supplemented with 0.5 μM 5-Iodoturbercidin (5-Itu), 0.5 μM AZD1152-HQPA (AZD) or 0.5 μM GSK1070916 (GSK). Control group was cultured in M2 medium supplemented with corresponding amount of DMSO.

### Oocyte fixation and immunofluorescent imaging

For the immunolocalization experiments, oocytes from wild type and *Sycp3* ^*−/−*^ mice were fixed in 1% PFA supplemented with 0.15% Triton X-100 as described in Kouznetsova et al. (2014). To get oocytes at the prometaphase II stage, germinal vesicle oocytes were matured at 37°C in M2 medium, and oocytes that had not underwent germinal vesicle breakdown after 2 h in culture were removed. Then oocytes were monitored every 1 h and those oocytes that showed the polar body extrusion were moved to a separate dish and fixed after additional 1 h 15 min in culture. To get oocytes at the metaphase II stage, oocytes were fixed within 1–2 h after isolation from the oviducts (18–20 h after the hCG injection). At this timepoint oocytes reach the metaphase II stage and remain arrested at this stage with chromosomes aligned at the spindle equator waiting for the fertilization (Kouznetsova et al., 2014). In inhibition assays, oocytes at the metaphase II stage were fixed 2 h after inhibitor addition. The antibodies used were human ACA (Antibodes Inc.) at a 1:100 dilution, rabbit anti-Aurora B (own production against peptide GLNTLSQRVLRKEPATTSALA) at 1:50 dilution, guinea pig anti-Aurora C (own production against peptide PGGELYKELQRHQKLDQQRT) at 1:50 dilution; guinea pig anti-Incenp (own production against peptide CTSYQMTPQGPKSIPK) at 1:50 dilution, anti-phospho-Aurora ABC (T2888/232/239) (Cell Signalling) at 1:50 dilution, and rabbit anti-phospho Hec1 (S55) (GeneTex) 1:50, rabbit anti-phospho-H3 (T3) (Abcam) at 1:50 dilution. The secondary antibodies were goat-anti-rabbit Alexa 488 (Invitrogen) at 1:1,000 dilution, goat-anti-guinea pig Alexa 555 1:1,000 and donkey-anti-human Alexa 647 (Invitrogen) 1:1,000. Oocytes were mounted in ProLong Gold (Thermo Fisher Scientific). Images were recorded with a Zeiss 900 Airyscan super-resolution microscope using a 63X/1.4NA oil immersion objective and post-processed in Imaris 9.4 software (Bitplane).

### Time-lapse imaging and centromere tracking

Low-resolution time-lapse imaging was performed on a wide-field Leica DMI6000 microscope with 20X/0.4NA HCX PL Fluotar dry objective (Leica). We imaged 30–40 sections z-sections spaced 1.5 mm at time interval of 10–15 min. High-resolution time-lapse imaging of oocytes for centromere tracking was performed using a Zeiss LSM 780 confocal microscope equipped with a 40X/1.2NA C-Apochromat water immersion objective (Carl Zeiss) using the 3D multi tracking macro (Rabut and Ellenberg, 2004). We imaged 17–19 consecutive z-confocal sections spaced 1.0 μm, at time intervals of 1.5–5 min. The temporal resolution allowed us to image 5–8 oocytes in the same experiment without apparent phototoxicity effects but also set a limitation for observing movements that lasts for less than 1.5–3 min. Centromere tracking was performed with Imaris image analysis software (Bitplane) using a tracking procedure as described previously (Kitajima et al., 2011; Kouznetsova et al., 2019). The full tracking procedure was performed for single chromatids and misaligned chromosomes only, chromosomes with centromeres that displayed weak 2mEGFP-CENP-C signal were excluded from the analysis. Data processing and plotting were performed using Fiji and MATLAB (MathWorks).

### Image analysis and statistical analysis

To create contour plots of average intensity distributions, we identified the positions of the centromeres in each oocyte as marked by ACA antibody using Fiji plugin ComDet (https://imagej.net/plugins/spots-colocalization-comdet). The areas around paired sister centromeres were cropped, rescaled so that the inter-centromere distance for all chromosomes become 100 pixels, and rotated to get the same horizontal orientation for all chromosomes, based on DAPI staining. For single chromatids, the images were rotated so that all chromatids were oriented vertically with the centromere on the top, based on DAPI staining. We excluded from analysis chromosomes and chromatids that were clustered and displayed signals from more than one chromosome or chromatid in the cropped area; damaged chromosomes/chromatids as seen by DAPI staining; chromosomes/chromatids that displayed non-specific signal in the cropped area, and chromosomes with inter-centromere distance bigger or smaller than 2 standard deviations from the mean. To obtain the final contour plots of the chromosomes, the protein distributions were summarized for all chromosomes in each oocyte, then these summarized distributions were averaged over all oocytes imaged in one experiment, and then over the experiments. To obtain the final contour plots of the chromatids, the protein distributions were summarized for all obtained chromatids. At least 3 independent experiments were performed to obtain each of the contour plots. To quantify pH3 signal, we measured the mean pH3 signal intensity on chromatin as outlined by DAPI, using Fiji software. The data was normalized to the average intensity observed in control oocytes for each experiment.

Quantitative data is presented as mean ± standard deviation, from at least three independent experiments. We used Student’s *t* test for comparisons between the two groups. The statistical analysis was performed by GraphPad Prism and a *P*-value of less than 0.05 was considered statistically significant. Image processing was performed with the help of Fiji, Adobe Photoshop, and Adobe Illustrator software.

## Results

### Distinct Aurora B/C pools are recruited to the kinetochore and inner central regions of MII chromosomes at the second meiotic division

**FIGURE 1:**
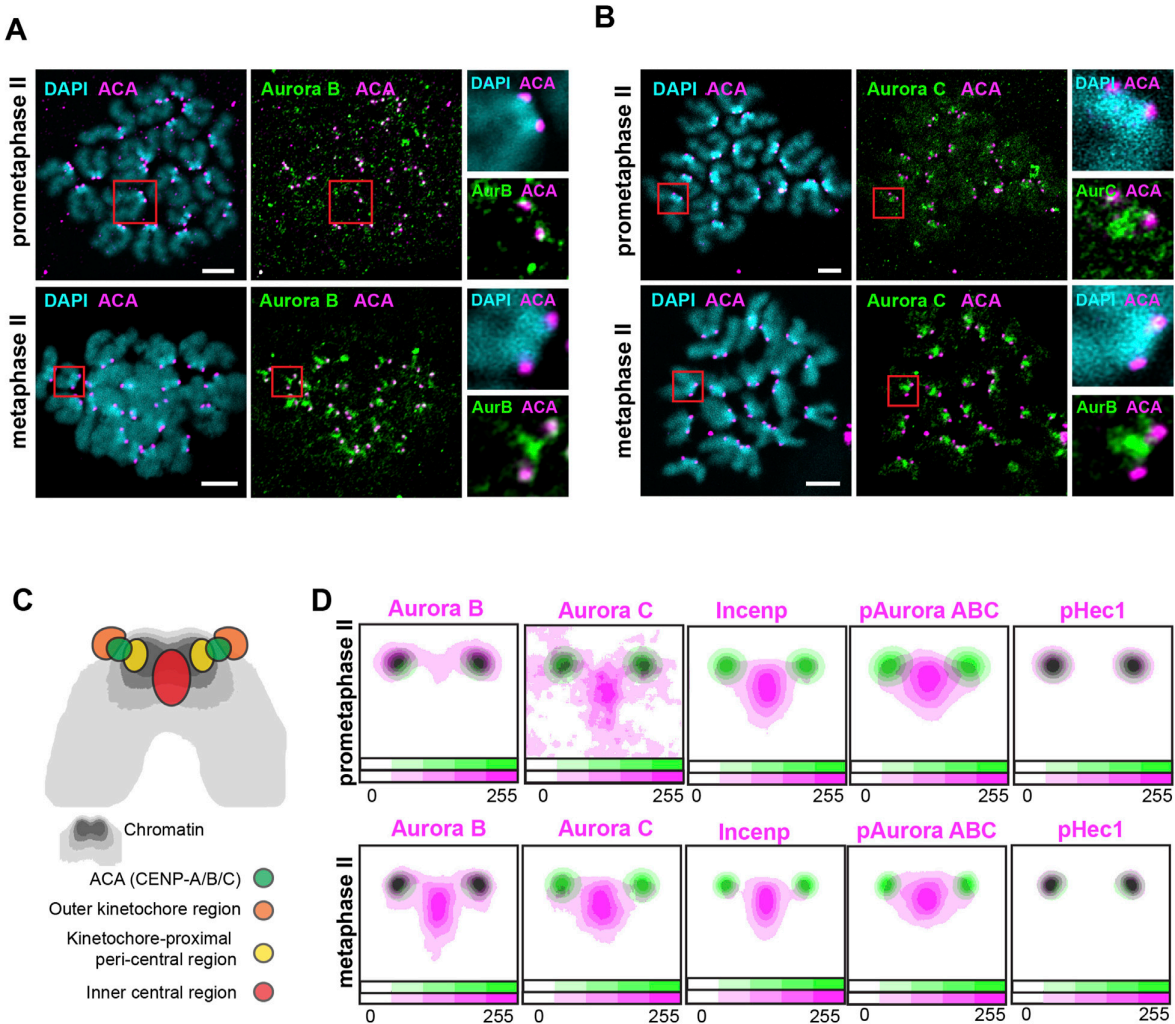
Aurora B, Aurora C, Incenp, pAurora ABC and pHec1 localize to discrete regions at prometaphase II and metaphase II. **(A, B)** Immunofluorescent images of the prometaphase II and metaphase II stage mouse oocytes stained with antibodies to AuroraB **(A)** or Aurora C **(B)** (green), ACA to label CENP-A/B/C (magenta) and DAPI to indicate chromosomes (cyan). Red squares indicate chromosomes shown enlarged in the inserts to the right. Bars, 5 μm. **(C)** A summary drawing of the centromere region at chromosomes at the second meiotic division. **(D)** Contour plots of averaged intensity distributions for Aurora B (n = 86/104 chromosomes a prometaphase II and metaphase II, respectively), Aurora C (n = 89/157), Incenp (n = 182/142), Aurora phosphorylated on T288/T232/T198 (pAurora ABC) (n = 102/138) and Hec1 phosphorylated on S55 (pHec1) (n = 69/80) (magenta) in relation to the position of CENP-A/B/C as defined by ACA aniserum (green) on chromosomes at prometaphase II and metaphase II. Intensities are color-coded, as indicated by the color bar at the bottom of each plot.

To characterize the processes involved in chromosome alignment and kinetochore-microtubule correction during the second meiotic division in mouse oocytes, we analyzed the localization of Aurora B and Aurora C at prometaphase II and metaphase II. Oocytes from young adult wild-type mice were fixed in PFA and stained with antibodies against Aurora B or Aurora C. The oocytes, co-stained with ACA (Anti-Centromeric Antiserum, recognizing centromere proteins CENP-A/B/C) and DAPI for chromosome visualization (see Figures 1A, B), were imaged using super-resolution microscopy.

The signals for Aurora B and Aurora C kinases show a certain variability within single each oocyte, as visualized in Supplementary Figures S1A, B, where all chromosomes from oocytes shown in Figures 1A, B are arranged side-by-side to facilitate comparison. To accurately determine and compare the localization of Aurora B, Aurora C and other proteins involved in correction of kinetochore-microtubule attachments at different stages, we averaged the signals from the centromeric zone across chromosomes from several independent experiments (see the scheme of the centromere zone in Supplementary Figure S2A). We then generated contour plots of protein intensities in relation to the positions of CENP-A/B/C proteins, as marked by ACA antiserum (refer to Materials and Methods for the details). We were able to define three regions in the centromere zone where we found Aurora B/C and related proteins, roughly corresponding to the regions found in mitotic cells: (i) an inner central region located between the sister centromeres (Figure 1C, red), corresponding to the inner centromere region described in mitotic cells, (ii) a region located at the periphery of the central pool proximal to the kinetochores (Figure 1C, yellow), corresponding to the outer-centromere kinetochore-proximal region, and (iii) an outer kinetochore region located outside of the CENP-A/B/C proteins marked by ACA antiserum (Figure 1C, orange).

We observed that Aurora B at prometaphase II was predominantly localized at the outer kinetochore region (Figures 1A, D). As oocytes progressed to metaphase II, Aurora B extended its localization to a chromatin region located between the sister chromatids (Figures 1A, D). Aurora C showed both kinetochore and central region localization at prometaphase II, but became almost exclusively localized to the central region at metaphase II (Figures 1B, D).

To further characterize the location of Aurora B and C kinases, we examined the localization of Incenp, a component of the chromosome passenger complex required for loading of Aurora B and Aurora C on chromosomes (Cairo and Lacefield, 2020). We observed a decreased amount of Incenp antibody signal at the kinetochore-proximal region at prometaphase II compared to the metaphase II stage (Figure 1D; Supplementary Figure S2B, see the variability of Incenp signal within a single oocyte in Supplementary Figure S1C). This observation supports the view that the localization of Aurora B/C changes from the kinetochore to the inner central region during the transition from prometaphase II to metaphase II. Notably, most of the Incenp signal is observed at the inner central region at both prometaphase II and metaphase II stages, while Aurora B is found at the outer kinetochore region. This suggests that at the second meiotic division Aurora B-Incenp complexes are much less abundant than compared to Aurora C-Incenp complexes.

We then set out to characterize the active fractions of Aurora B and C. We utilized a pAurora ABC antibody, which identifies a conserved phosphorylation site on the kinase domain across all Aurora isoforms (pT288/pT232/pT198 for Aurora A, B, and C respectively), important for the full kinase activity (Yasui et al., 2004; Shrestha et al., 2017). Given that Aurora A is located near the spindle poles, labelling observed by the pAurora ABC antibody on chromosomes identifies active Aurora B/C (Nguyen and Schindler, 2017). We observed that the pAurora ABC signal was predominantly found at the inner central region and kinetochore-proximal peri-central regions during both prometaphase II and metaphase II stages (Figure 1D; Supplementary Figure S2C, see the variability of pAurora ABC signal within a single oocyte in Supplementary Figure S1D). This indicates and Aurora B located at the kinetochores only contributes minimally to the overall Aurora B/C kinase activity. Using an antibody that specifically detects active Aurora C (phosphorylated on T171), we identified active Aurora C at the inner central region and the kinetochore-proximal peri-central region during prometaphase II. Furthermore, an increased fraction of pAurora C was found on the inner central region at metaphase II stage (Supplementary Figures S2D, E), indicating the shift in the Aurora C activity towards the inner central region from prometaphase II to the metaphase II stages. It is noteworthy that a minor fraction of Incenp, pAurora ABC and pAurora C is present on the kinetochores during prometaphase II and metaphase II (Supplementary Figure S2F) (revealed by overexposure). The functional role for Aurora B/C at the kinetochores was analyzed by examining the phosphorylation status of Aurora B/C at outer kinetochore targets. We used an antibody against Hec1 phosphorylated at S55 (a modification crucial for destabilizing kinetochore-microtubule attachments) (DeLuca et al., 2011), and found pHec1to be present on the outer kinetochores at prometaphase II and metaphase II (Figure 1D; Supplementary Figure S2G, see the variability of pHec1 signal within a single oocyte in Supplementary Figure S1E), supporting the presence of active Aurora B/C at the outer kinetochore region.

In summary, during the second meiotic division Aurora B and Aurora C kinases are found at the kinetochore and inner central region, accumulating at the inner central region at the metaphase II stage. Notably, unlike mitosis, these regions form a triangular pattern at the prometaphase and metaphase stages rather than a straight line. The vast majority of enzymatically active Aurora B/C kinases are found at the inner central region separated from the kinetochore targets at the prometaphase II and metaphase II stages. This is different from the situation in mitotic cells, where active Aurora B phosphorylated on T232, is found mostly at the outer kinetochores at prometaphase and then become localized to the inner centromere region at the metaphase stage (Broad et al., 2020). It is interesting that unlike mitosis, kinetochore proteins during meiosis II remain phosphorylated upon chromosome alignment at the metaphase plate. The presence of Aurora B kinase at the outer kinetochore and inner central regions in meiosis II cells suggests a dual role in phosphorylating proteins both at the outer kinetochore and at the inner central region at the metaphase II stage. The abundant localization of Aurora C to the inner central region at metaphase II indicates that primary function for Aurora C is likely coupled to this region. However, it cannot be excluded that Aurora C also is involved in the phosphorylation of kinetochore targets at the outer kinetochore domain, together with Aurora B.

### Haspin inhibition leads to loss of histone H3 phosphorylation at the inner central region and disappearance of the central Aurora B/C kinase pool from metaphase II chromosomes

**FIGURE 2:**
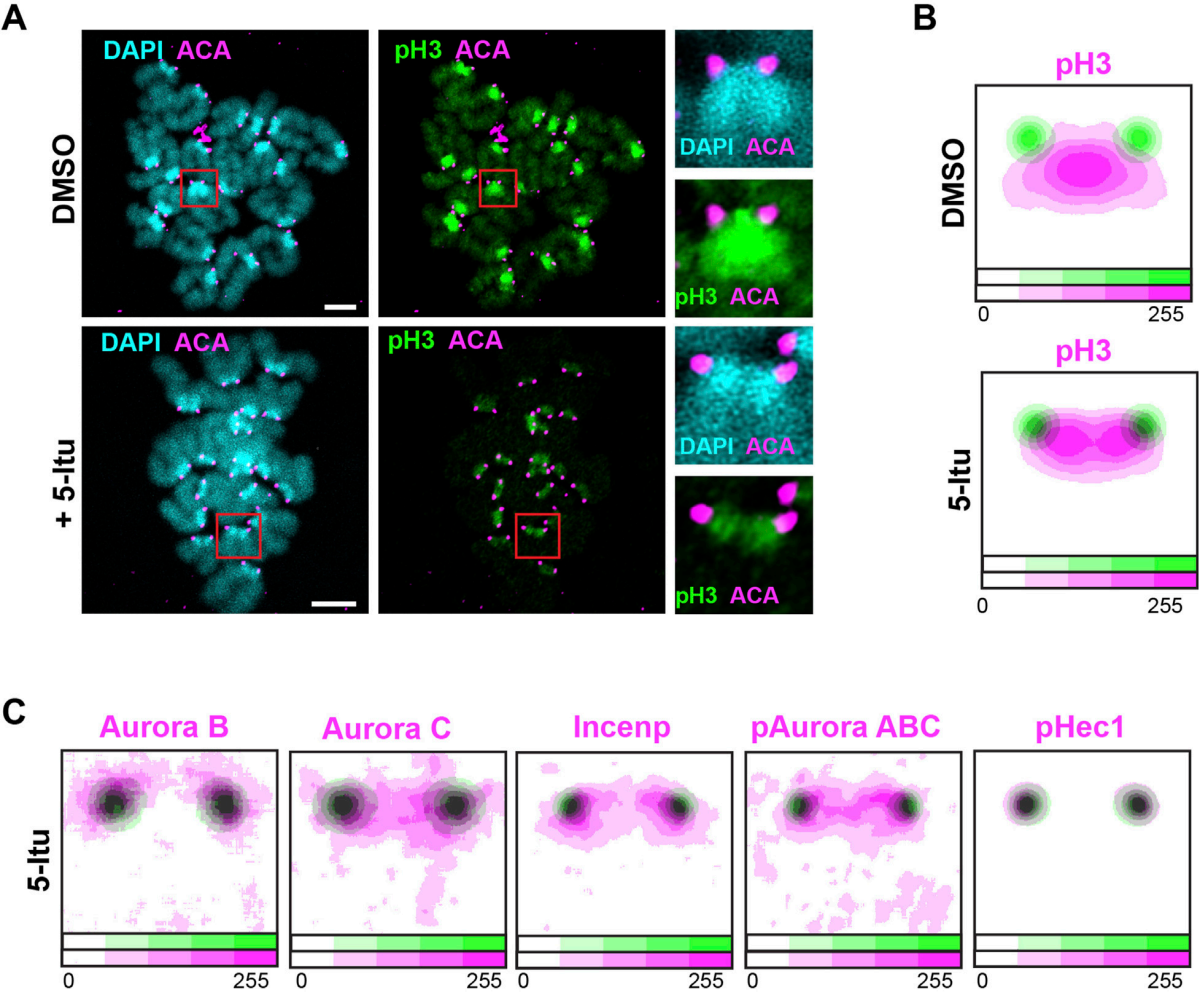
The central pool of Aurora B/C kinases is dependent on histone H3 phosphorylation by haspin kinase. **(A)** Immunofluorescent images of metaphase II stage mouse oocytes stained with antibodies against histone H3 phosphorylated on T3 (pH3, green) and ACA to label CENP-A/B/C (magenta). DAPI was used to indicate chromosomes (cyan). The oocytes were incubated with DMSO (upper row) or with the histone H3 inhibitor 5-Iodotubercidin (5-Itu). Red squares indicate chromosomes that are shown enlarged in the inserts to the right. Bars, 5 μm. **(B)** Contour plots of averaged intensity distributions for pH3 in magenta incubated with DMSO or with the histone H3 inhibitor 5-Itu, shown in relation to the position of CENP-A/B/C as defined by ACA antiserum (green). n = 206/120 for the DMSO- and 5-Itu-incubated chromosomes, respectively. Intensities are color-coded, as indicated by the color bar at the bottom of the plots. **(C)** Contour plots of averaged intensity distributions for Aurora B (n = 55), Aurora C (n = 54), Incenp (n = 129), Aurora phosphorylated at T288/T232/T198 (pAurora ABC) (n = 75) and Hec1 phosphorylated at S55 (pHec1) (n = 145) shown in magenta in relation to the position of CENP-A/B/C, as defined by ACA antiserum (green), in metaphase II oocyte in presence of 5-Itu. Intensities are color-coded, as indicated by the color bar at the bottom of the plots.

The localization of Aurora B/C to the inner central region is dependent on phosphorylation of histone H3 on T3 by haspin kinase during mitosis, meiosis I and II in spermatocytes and meiosis I in oocytes (Nguyen et al., 2014; Quartuccio et al., 2017; Broad et al., 2020; Inés Berenguer et al., 2022). We found by using super-resolution immunofluorescent imaging that an antibody that detects histone H3 phosphorylation on T3 (pH3) labels the inner central region of sister chromatids in mouse metaphase II oocytes (Figures 2A, B). Following haspin inhibition by 5-Iodotubercidin (5-Itu), the pH3 signal became weaker (Supplementary Figure S3A) and the staining pattern for pH3 changed from labelling the inner central region to labelling the peri-central kinetochore-proximal region in mouse metaphase II oocytes (Figures 2A, B). Interestingly, the inter-kinetochore distance remains unchanged, suggesting that loss of haspin activity does not affect spindle tension (Supplementary Figure S3B). Aurora B and C kinases exhibit distinct localization patterns upon inhibition of the haspin-pH3 loading pathway: Aurora B signal relocates to the outer kinetochore region, while Aurora C localization shifts to the periphery of the central region (Figure 2C; Supplementary Figure S3C). Incenp was also found at the peri-central and outer kinetochore region upon haspin inhibition, along with the active form of Auroras, pAurora ABC (Figure 2C; Supplementary Figure S3C). Notably, pHec1 maintains its localization on the outer kinetochores, suggesting that its phosphorylation status is supported by kinetochore-bound Aurora B/C kinases that are not affected by haspin kinase inactivation (Figure 2C; Supplementary Figure S3C). The change in localization of Aurora B/C kinases in the presence of 5-Itu cause loss of chromosome alignment at the metaphase II stage, as revealed by time-lapse imaging of mouse oocytes (Supplementary Figures S3D, E).

In conclusion, haspin inhibition in mouse oocytes leads to a loss of the central pool of Aurora B/C kinases and chromosome misalignments at the metaphase II stage. This suggests that the inner central pool of Aurora B/C kinases plays a crucial role in maintaining chromosome alignment at the metaphase II stage.

### Single chromatids show an increased level of susceptibility to misalignments during metaphase II upon Aurora B/C inhibition

To further investigate the role the inner central region between sister chromatids at the second meiotic division, we examined single chromatids. Single chromatids differ from normal chromosomes at the as they lack a paired sister chromatid. We used oocytes derived from Sycp3 KO mice, which display a few single chromatids in each oocyte at the metaphase II stage (Yuan et al., 2002). Loss of Sycp3 proteins contributes to segregation errors during meiosis I but does not have functional role at the second meiotic division in oocytes (Kouznetsova et al., 2005).

**FIGURE 3:**
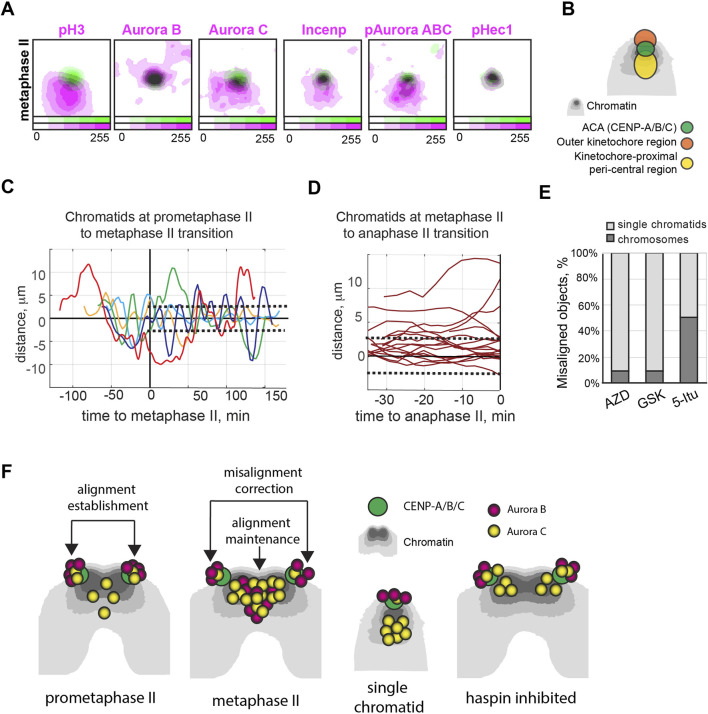
Single chromatids lack the central pool of Aurora B/C and is susceptible to Aurora B/C inhibition. **(A)** Contour plots of averaged intensity distributions for pH3 (n = 15), Aurora B (n = 14), Aurora C (n = 13), Incenp (n = 12), pAurora ABC (n = 7) and pHec1(n = 6) (magenta) in relation to the position of CENP-A/B/C as defined by ACA antiserum (green) for single chromatids at the metaphase II stage in mouse oocytes. Intensities are color-coded, as indicated by the color bar at the bottom of the plots. **(B)** A summary drawing of the centromere region at single chromatids at the second meiotic division. **(C, D)** Changes in the distance from the centromere to the spindle equator plane (on the vertical axis) for 5 single chromatids at the transition from prometaphase II to metaphase II (**(C)**, each chromatid is color-coded) or 15 single chromatids at the transition from metaphase II to anaphase II (**(D)**, red). Time (on the horizontal axis) is shown in relation to the onset of the metaphase II **(C)** or anaphase II **(D)**. Dotted lines indicate the average thickness of the metaphase plate at the metaphase II. **(E)** The percentage of chromosomes (dark grey) and single chromatids (light grey) among the first five objects to misalign in the presence of Aurora B inhibitor AZD1152-HQPA (AZD, n = 10); Aurora B/C inhibitor GSK1070916(GSK, n = 10) or haspin inhibitor 5-Iodotubercidin (5-Itu, n = 59). **(F)** Summary drawings for Aurora B and Aurora C localization on mouse chromosomes and single chromatids at the second meiotic division and after haspin inhibition.

We stained oocytes from young adult Sycp3 KO mice with an antibody recognizing pH3. The localization plot confirmed that single chromatids lack the inner central region, as defined by histone H3 phosphorylation (, Fig. Supplementary Figures S3, 4A, see Figure 3B for region scheme in single chromatids). Single chromatids also lacked Aurora B, Aurora C, Incenp, and pAurora ABC localization to the inner central region. Notably, Aurora B show an outer kinetochore localization at single chromatids, while Aurora C, Incenp and active forms of Auroras recognized by pAurora ABC antibody retained peri-central region localization (Figure 3A; Supplementary Figure S4A). pHec1 was found on the kinetochores, validating the activity of Aurora B/C at this location on single chromatids (Figure 3A; Supplementary Figure S4B). Generally, the localization of the analyzed proteins on single chromatids closely resembled the patterns seen for chromosomes following inhibition of haspin (compare Figures 3A, 2C).

How will the absence of the inner central region in single chromatids affect their dynamics during the second meiotic division? To analyze this, we labelled the centromeres of oocytes from Sypc3 KO females with EGFP-Cenp-C and chromosomes with H2B-mCherry. We then performed time-lapse imaging at intervals of 2.5–5 min from the onset of anaphase I until the first 1.5–2 h of metaphase II arrest. The presence of single chromatids in oocytes had no effect on temporal progression from anaphase I to metaphase II arrest, when compared to oocytes with a normal chromosome complement (Supplementary Figure S4C, compared to Kouznetsova et al. (2019)). At the prometaphase II stage, single chromatids were not delayed near the spindle pole, and each crossed the equator plane at least once (Figure 3C), suggesting proficiency for congression to the metaphase plate in the absence of a central pool of Aurora B/C kinase. At the metaphase II stage, single chromatids either oscillated between the poles or remained stable within the metaphase plate (Figure 3D). Aurora B/C activity at the outer kinetochores (as shown by the strong staining of pHec1) likely contributes to the competence of single chromatids to realign at the metaphase plate. The predominant outer kinetochore localization of Aurora B but not Aurora C (Figure 3A) suggests an important role for this kinase in promoting re-alignment of single chromatids to the spindle equator at metaphase II.

We next investigated how inhibition of Aurora B/C or depletion of the two kinases from the inner central region influence the dynamics of chromosomes and chromatids during metaphase II. We tracked the dynamics of chromosomes and single chromatids in live oocytes from Sycp3 KO mice, with centromeres labeled with Cenp-C-EGFP and chromosomes with H2B-mCherry. We used AZD1152-HQPA, a primary Aurora B kinase inhibitor; GSK1070916, a compound that inhibits both Aurora B and C activity (Anderson et al., 2009; Cee et al., 2010); and the haspin inhibitor 5-Itu to deplete both Aurora B and Aurora C at the inner central region. We then monitored chromosome and chromatid misalignments at the metaphase II stage for 2 h following addition of the inhibitors.

When Aurora B/C kinase activity was reduced in metaphase II oocytes from Sycp3 KO mice using the chemical inhibitors, a majority of the first recorded misaligned objects was represented by single chromatids (Figure 3E). Given that single chromatids lack an inner central region and a corresponding Aurora B/C pool, this confirms an active role for Aurora B/C at the outer kinetochore in modifying microtubule-kinetochore attachments during metaphase II. We suggest that the absence of Aurora B/C at the inner central region makes single chromatids more prone to misalignment. Our hypothesis further implies that depletion of Aurora B/C through haspin inhibition at the inner central region of chromosomes should result in misalignment of chromosomes at the metaphase plate at a rate comparable to single chromatids. Consistent with this, tracking of chromosome and single chromatid centromeres in the presence of the haspin inhibitor 5-Itu revealed an approximately equal number of chromosomes and chromatids among the misaligned objects 2 h after inhibitor addition (Figure 3E). This observation supports the hypothesis that the primary function of Aurora B/C kinases at the inner central region is to maintain chromosome alignment at the metaphase II plate.

## Discussion

The regulation of kinetochore-microtubule attachments in mitosis requires phosphorylation of substrates at outer kinetochores by Aurora B. However, Aurora B kinase is primarily located at the middle of a line connecting two sister kinetochores in mitotic cells (Broad et al., 2020; Liang et al., 2020). To reconcile the location of the kinase with its regulatory role at the outer kinetochore, an “Aurora gradient” model suggests that Aurora B kinase emanates a diffusible gradient to phosphorylate its substrates at the kinetochores (Liu et al., 2009; Uchida et al., 2009). At prometaphase, when inter-kinetochore tension is low and attachments need to be corrected, kinetochores are within the reach of Aurora B kinase activity. However, at metaphase, as tension increases, kinetochores are pulled away from the centrally localized Aurora B, preventing phosphorylation of the kinetochore targets and thus stabilizing the correct kinetochore-microtubule attachments. This model provides a good explanation for kinetochore-microtubule dynamics in mitosis, where inter-kinetochore distances are small (about 1 µm) and kinetochores and Aurora B are located on the same line.

It was recently shown Aurora B is recruited to the kinetochores and to the inner region by two separate pathways (Broad et al., 2020; Liang et al., 2020; Cairo et al., 2023). A “direct recruitment” model proposes that the kinetochore fraction phosphorylates the kinetochore substrates at prometaphase but its activity is lost when tension increases and the kinetochore architecture changes at metaphase, thus stabilizing the kinetochore-microtubule attachments. The other fraction is recruited to the central region and is not directly coupled to the Aurora B activity at the kinetochores (Broad and DeLuca, 2020).

In this study, we explored the localization and activity of Aurora B and Aurora C in mouse oocytes during the second meiotic division. We observed that the inner central region and the sister kinetochores form a pattern that resembles a triangle in mouse oocytes, rather than a straight line as seen in mitotic cells (Figure 1, scheme in Figure 3F). Notably, the triangular configuration of the central region and kinetochores at the second meiotic division is also observed in human oocytes (Avo Santos et al., 2011) and therefore not a result related to the acrocentric structure of mouse chromosomes. Considering that the inter-kinetochore distance in mouse oocytes is approximately 2 μm, it would be difficult for centrally located Aurora B and C to access its kinetochore targets; additionally, a triangular shape is not optimal for gradient formation. Therefore, our experimental data does not support a gradient model for meiosis II chromosomes.

On the other hand, we found that Aurora B change localization pattern from being predominantly at the kinetochore at prometaphase II to then be seen to accumulate at the inner central region at metaphase II (Figure 1A), in agreement with a direct recruitment model as proposed for mitotic cells. In contrast, however, to mitotic cells a significant portion of Aurora B in meiosis II cells remain at kinetochores also at metaphase II (Figures 1A, C, scheme in Figure 3F), ensuring that that kinetochore targets during meiosis II (e.g., Hec1, Figure 1C) remain phosphorylated at the metaphase II stage. The presence of active Aurora B at kinetochores is likely to be of importance to correct errors in kinetochore-microtubule alignments during the prolonged metaphase II stage in oocytes. Unlike Aurora B, Aurora C exhibits a relatively weak intensity at the outer kinetochore region at the prometaphase II stage, with the majority of Aurora C concentrated at the inner central region (Figures 1B, C, scheme in Figure 3F). The distinct localization patterns for Aurora B and Aurora C corroborates separate roles for the two kinases during meiosis (Balboula and Schindler, 2014; Nguyen et al., 2018).

Analysis of the localization of Incenp and the enzymatically active form of Aurora B/C, pAurora ABC, reveals that the major active fraction of Aurora B/C is situated at the inner central region of the metaphase II chromosomes. Experiments with single chromatids, which lack this central pool of Aurora B/C (Figure 3A, scheme in Figure 3F), demonstrate that this pool is not essential for initial congression of single chromatids to the metaphase plate during prometaphase II, nor for the realignment of sister chromatids to the metaphase plate during metaphase II. Consequently, we propose that the minor kinetochore-bound fraction of Aurora B/C primarily drives the dynamic corrections of kinetochore-microtubule attachments necessary for alignment to the metaphase plate. The function for the kinetochore fraction of Aurora B/C seems to be conserved, as also mitotic chromosomes that lack the central fraction of Aurora B still were competent for chromosome alignment at the prometaphase stage (Liang et al., 2020).

Inhibition of haspin in mouse oocytes leads to a loss of the central Aurora B/C pool (Figure 3A and scheme in Figure 3F), resulting in chromosome misalignments (Supplementary Figure S3E, also reported in Cairo et al. (2023)), indicating a role for the centrally located Aurora B/C kinases in maintaining chromosome alignment at the metaphase plate. Importantly, we found that single chromatids that lack the inner central Aurora B/C pool are more susceptible to loss of Aurora B/C kinase activity than chromosomes (Figure 3E), supporting critical importance of centrally located Aurora B/C for the alignment maintenance.

The exact mechanism how this Aurora B/C pool aids in alignment is unclear. Aurora B/C at the inner central region encompasses the majority of the enzymatically active Aurora B/C, implying a kinase-dependent role in sustaining chromosome alignment during the metaphase II stage. We hypothesize that the central Aurora B/C pool, transported to the spindle equator by chromosomes, aids in forming the central spindle region during metaphase, resembling an important role of Aurora B in midzone formation during anaphase shown in mitotic cells (Ali and Stukenberg, 2023). Indeed, Aurora B inhibition disrupts the formation of the anti-parallel microtubule bundles at the central spindle region in mitotic cells, and displacing Aurora B from the inner centromere region using a CENP-B-INCENP-GFP construct displaced the cross-linking protein PRC1 from the spindle’s central region (Matković et al., 2022).

In conclusion, our study reveals that Aurora B/C kinase fractions at kinetochore and the inner central region collaboratively maintain chromosomal alignment to prevent aneuploidy and missegregation during metaphase II. These findings are in agreement with previous studies suggesting that both pools work together for error correction during mouse oogenesis (Cairo et al., 2023). The major central Aurora B/C pool stabilizes chromosome alignment during metaphase II arrest, while the minor kinetochore fraction aids in chromosome realignment.

## Data Availability

The raw data supporting the conclusions of this article will be made available by the authors, without undue reservation.
