# Supplementary material for: Aurora B and Aurora C pools at two chromosomal regions collaboratively maintain chromosome alignment and prevent aneuploidy at the second meiotic division in mammalian oocytes
Source: Front Cell Dev Biol. 2024 Sep 17;12:1470981. doi: 10.3389/fcell.2024.1470981 (PMC11442388; doi:10.3389/fcell.2024.1470981)
Supplement: Supplementary file 1 [file Image4.pdf]

Figure S4

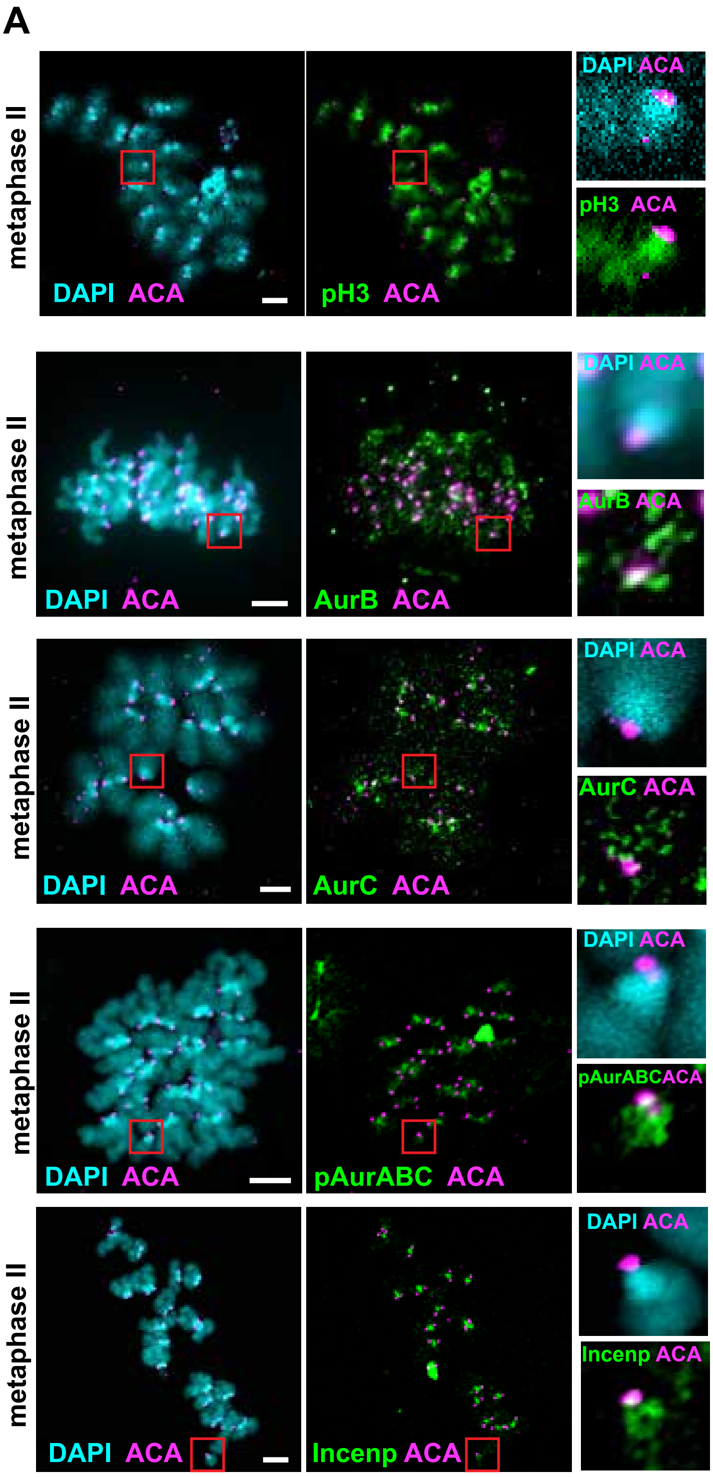
A
metaphase II
DAPI ACA
pH3 ACA
AurB ACA
AurC ACA
pAurABC ACA
pAurABCACA
Incenp ACA
B
pHec1 ACA
C
Progress from anaphase I to metaphase II
oocytes
0
100
200
time from anaphase I onset, min
completion of MI
interkinesis
prometaphase II
metaphase II
