# Supplementary figures and images for: Aurora B and Aurora C pools at two chromosomal regions collaboratively maintain chromosome alignment and prevent aneuploidy at the second meiotic division in mammalian oocytes
Source: Front Cell Dev Biol. 2024 Sep 17;12:1470981. doi: 10.3389/fcell.2024.1470981 (PMC11442388; doi:10.3389/fcell.2024.1470981)

Figure S2

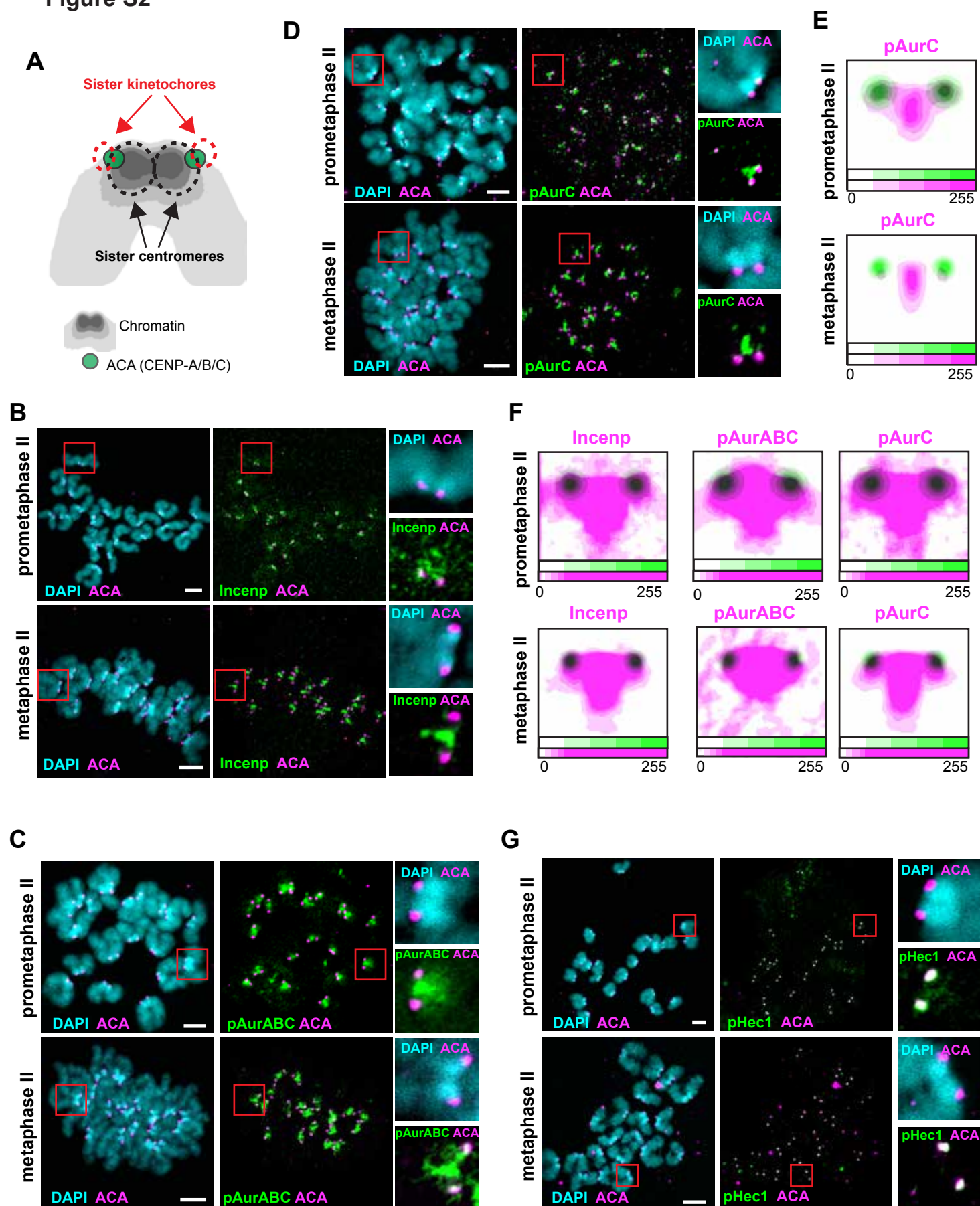

Supplement: Supplementary file 2 [file Image2.pdf]

Figure S3

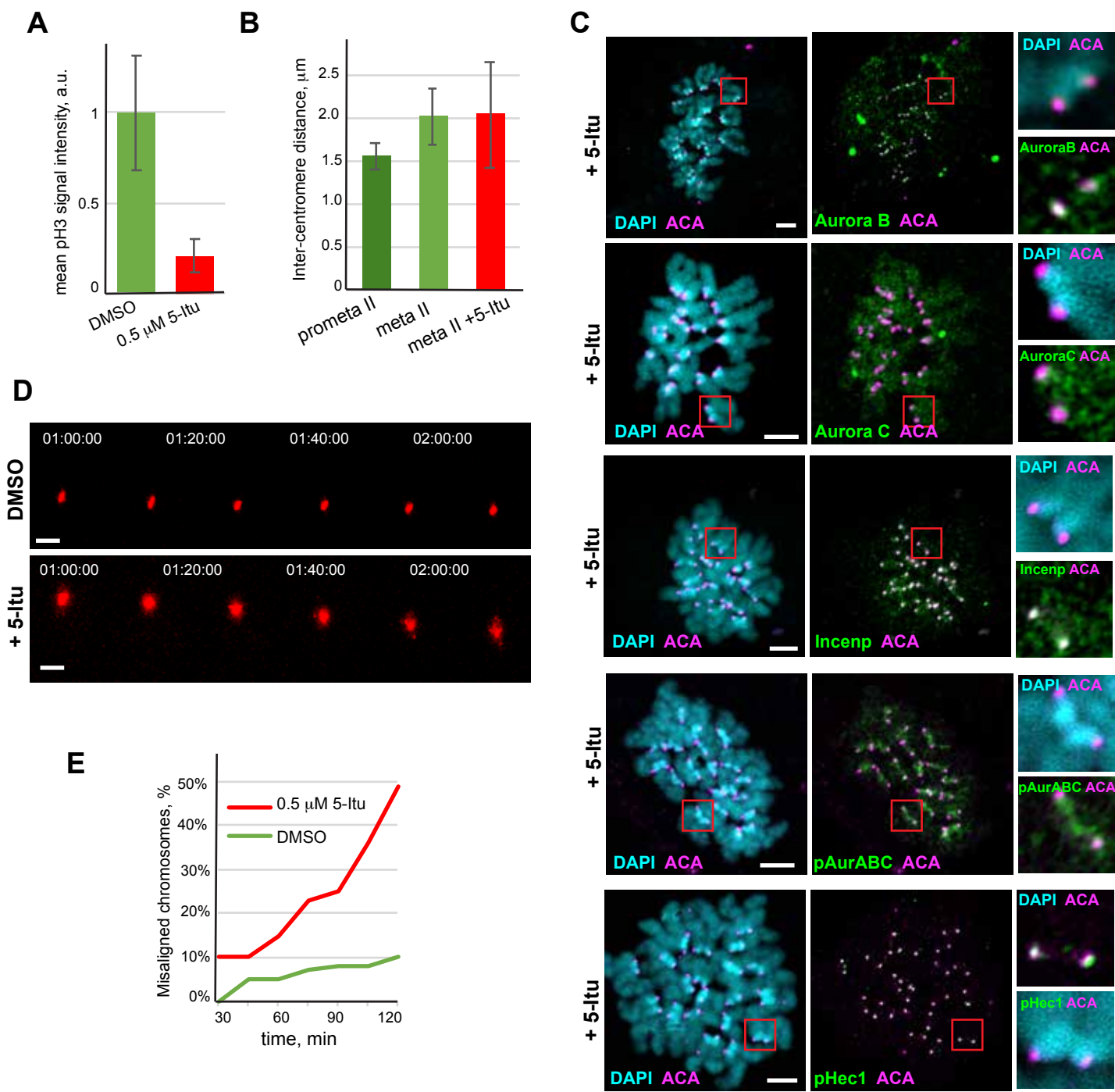

Supplement: Supplementary file 3 [file Image3.pdf]

Figure S1

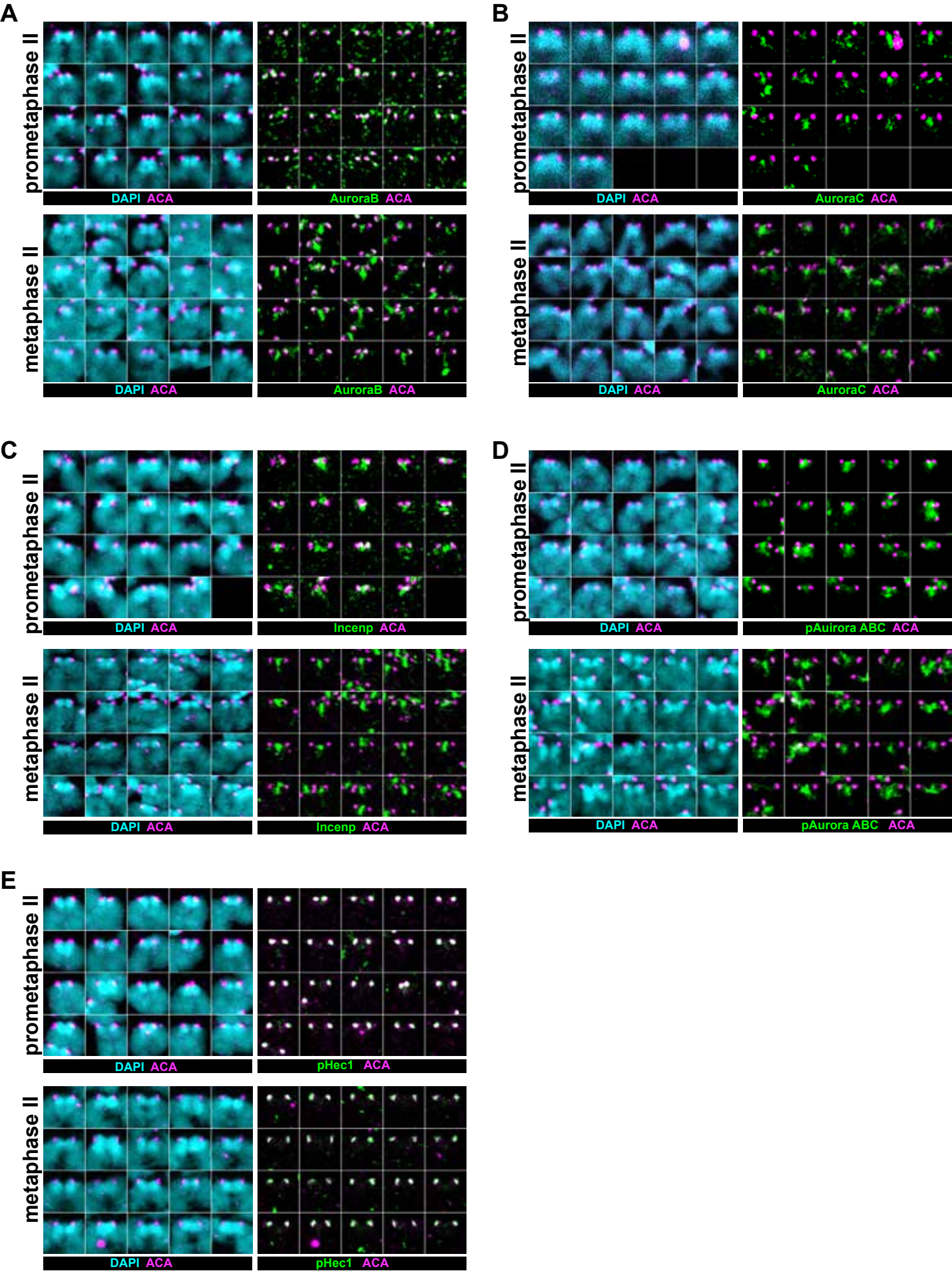

Supplement: Supplementary file 5 [file Image1.pdf]
