# Supplementary material for: Aurora B and Aurora C pools at two chromosomal regions collaboratively maintain chromosome alignment and prevent aneuploidy at the second meiotic division in mammalian oocytes
Source: Front Cell Dev Biol. 2024 Sep 17;12:1470981. doi: 10.3389/fcell.2024.1470981 (PMC11442388; doi:10.3389/fcell.2024.1470981)
Supplement: Supplementary file 4 [file DataSheet1.PDF]

Supplementary Figure Legends

Supplementary Figure 1.

(A-E) Variability in the antibody staining within a single oocytes at the prometaphase II and metaphase II stages for Aurora B (**A**), Aurora C (**B**), Incenp (**C**), Aurora phosphorylated at T288/T232/T198 (pAurora ABC) **(D)** and Hec1 phosphorylated at S55 (pHec1)(**E**) (green). ACA was used to label CENP-A/B/C (magenta) and DAPI to indicate chromosomes (cyan). All chromosomes found on images presented on Fig. 1A,B, Fig.2SB, C, G are cropped and arranged side-by side to facilitate comparison. Crop size is 5μm for all images.

Supplementary Figure 2.

(A) A schematic representation of the positions of centromeres and kinetochores in mouse MII oocytes. MII chromosome comprises paired sister chromatids held together by cohesion. The sister centromeres (black dotted circles) are defined by the presence of compacted chromatin (darker grey). The sister kinetochores (red dotted circles) are located just outside the CENP-A/B/C proteins, as recognized by ACA antiserum (green dots).

(B, C, D, G) Immunofluorescent images of prometaphase II and metaphase II mouse oocytes stained with antibodies to Incenp (**B**), Aurora phosphorylated at T288/T232/T198 (pAurora ABC) (**C**), Aurora C phosphorylated at T171 (pAurC) (**D**) and Hec1 phosphorylated at S55 (pHec1)(**G**) (green). ACA was used to label CENP-A/B/C (magenta) and DAPI to indicate chromosomes (cyan). Red squares indicate chromosomes shown enlarged in the inserts to the right. Bars, 5μm.

(E) Contour plots of averaged intensity distributions for Aurora C phosphorylated at T171 (pAurC) (n=123/163 chromosomes a prometaphase II and metaphase II, respectively) (magenta) in relation to the position of CENP-A/B/C as defined by ACA aniserum (green) on chromosomes at prometaphase II and metaphase II. Intensities are color-coded, as indicated by the color bar at the bottom of each plot.

(F) Contour plots of averaged intensity distributions for Incenp, pAurABC and pAurC (magenta) on chromosomes at prometaphase II and metaphase II, shown in Fig.1D, with a different color scheme, highliting the minor fraction of the proteins observed at the kinetochore region. CENP-A/B/C positions are defined by ACA aniserum (green). Intensities are color-coded, as indicated by the color bar at the bottom of each plot.

Supplementary Figure 3

(A) Mean intensity of the pH3 signal on chromatin in metaphase II oocytes decreases in the presence of 0.5μM of 5-Itu in comparison to control oocytes incubated in DMSO only ($p<0.001$, Students's t-test). Data presented at mean ± std, n=11, 13 for control oocytes and in the presence of 5-Itu, repectively. a.u. - arbitrary units.

(B) The distance between sister centromeres increases from prometaphase to metaphase II stages, but does not change in the presence of 0.5 μM 5-Itu (n=110, 160, 80 for prometaphase II, metaphase II and metaphase II in the presence of 5-Itu).

(C) Immunofluorescent images of the metaphase II stage mouse oocytes incubated in the presence of 0.5 mM 5-Itu, stained with antibodies to Aurora B, Aurora C, Incenp, Aurora phosphorylated at T288/T232/T198 (pAurora ABC) and Hec1 phosphorylated at S55 (pHec1) (green), ACA to label CENP-A/B/C (magenta) and DAPI to indicate chromosomes (cyan). Red squares indicate chromosomes shown enlarged in the inserts to the right. Bars, 5μm.

(D, E) Chromosomes drift from the metaphase plate in the presence of 0.5 mM 5-Itu inhibitor of haspin kinase. (**D**) Examples of time-lapse imaging of live oocytes in the presence of DMSO only or 0.5mM 5-Itu, where chromosomes are labelled by H2B-mCherry (red). Bar, 10mm. **(E)**

Percentage of misaligned chromosomes in the presence of 5-Itu (red) and in control oocytes (DMSO, green) over the first 2 hours after inhibitor addition.

Supplementary Figure 4

(A) Immunofluorescent images of the metaphase II stage mouse oocytes displaying single chromatids, stained with antibodies to histone H3 phosphorylated on T3 (pH3), Aurora B, Aurora C, Incenp, Aurora phosphorylated at T288/T232/T198 (pAurora ABC) and Hec1 phosphorylated at S55 (pHec1) (green), ACA to label CENP-A/B/C (magenta) and DAPI to indicate chromosomes (cyan). Red squares indicate single chromstids shown enlarged in the inserts to the right. Bars, 5μm.

(B) Progress from the prometaphase II to metaphase II stages in oocytes displaying single chromatids (n=6). The time is shown on the horizontal axis in min from the anaphase II onset, the stages are color-coded as indicated at the bottom.
